# Microarray Selection of Cooperative Peptides for Modulating Enzyme Activities

**DOI:** 10.3390/microarrays6020008

**Published:** 2017-04-26

**Authors:** Jinglin Fu

**Affiliations:** 1 Department of Chemistry, Rutgers University-Camden, Camden, NJ 08102, USA; jinglin.fu@rutgers.edu; Tel.: +1-856-225-6612; 2 Center for Computational and Integrative Biology, Rutgers University-Camden, Camden, NJ 08102, USA

**Keywords:** microarray, peptides, enzyme inhibition, β-galactosidase

## Abstract

Recently, peptide microarrays have been used to distinguish proteins, antibodies, viruses, and bacteria based on their binding to random sequence peptides. We reported on the use of peptide arrays to identify enzyme modulators that involve screening an array of 10,000 defined and addressable peptides on a microarray. Primary peptides were first selected to inhibit the enzyme at low μM concentrations. Then, new peptides were found to only bind strongly with the enzyme–inhibitor complex, but not the native enzyme. These new peptides served as secondary inhibitors that enhanced the inhibition of the enzyme together with the primary peptides. Without the primary peptides, the secondary effect peptides had little effect on the enzyme activity. Conversely, we also selected peptides that recovered the activities of inhibited enzyme–peptide complex. The selection of cooperative peptide pairs will provide a versatile toolkit for modulating enzyme functions, which may potentially be applied to drug discovery and biocatalysis.

## 1. Introduction

Small molecules that regulate enzyme activity play an important role in many biological functions, and are crucial for drug discovery [1,2]. Screening libraries of small molecules, peptides, and nucleic acids has been widely used to discover ligands that bind to proteins and modulate their functions [3,4]. Peptides represent a promising class of potential enzyme modulators [5] due to their large chemical diversity [6] and the existence of well-established approaches for library synthesis [7]. Peptides and their derivatives are found to inhibit many important enzymes [8], such as dehydrogenases [9], protein kinases [10], and proteases [11]. Cell-permeable peptides are becoming more and more useful in blocking cellular signaling pathways [12,13]. Over the past few decades, peptide microarrays have been developed for the high-throughput screening of a library of peptides that can bind to biological targets and alter their functions [7]. These arrays can be made by printing pre-synthesized peptides [14], SPOT synthesis (spotting and synthesis) [15], and light-directed, spatially-addressable synthesis [16]. Hydrogel-coated peptide microarrays were also reported as a means of screening for enzyme activity that is modulated by specific protein–peptide interactions, and has made it possible to perform activity assays using high-density microarrays [17].

Here, we reported on a selection of cooperative peptide pairs for inhibiting enzymes by screening a library of peptides specifically for binding to the inhibited enzyme complex on a microarray. Using this approach, we selected new peptides that enhanced the inhibition of the target enzyme by using them together with a primary inhibitory peptide. Without the primary peptide, these new peptides showed little inhibition impact on the enzyme activity. We also demonstrated that some negatively charged peptides could recover the activity of inhibited peptide/enzyme complex.

## 2. Materials and Approach

### 2.1. Materials

Fluorescein di-β-d-galactopyranoside (FDG), resorufin β-d-galactopyranoside (RBG), and Alexa Fluor 647 (Alexa 647) were purchased from Invitrogen (Eugene, OR, USA). Phenylethyl β-d-thiogalactoside (PETG), β-galactosidase (β-Gal), phosphate buffered saline (PBS), tris buffered saline (TBS), and formaldehyde were obtained from Sigma (St. Louis, MO, USA). A 4 mg/mL stock solution of β-Gal was prepared in 10 mM potassium phosphate buffer with 0.1 mM MgCl_2_ at pH 7.4. A 2 mg/mL stock solution of peptide was first prepared in pure water, and then diluted in phosphate buffer to the desired concentration. Custom peptides were purchased from Sigma.

### 2.2. Microarray Fabrication

Microarrays of 10,000 20-mer peptides were produced by Arizona State University as previously reported [14,17]. Briefly, 10,000 distinct polypeptide sequences were spotted in duplicate onto a glass slide possessing an amino-silane surface coating. Each polypeptide was 20 residues in length, with 17 positions randomly chosen from a set of 19 amino acids (excluding cysteine). The C-terminal 3 positions of each peptide was a glycine–serine–cysteine (GSC) linker, which was used for conjugating peptides to amino-silane surfaces via a maleimide linker, sulfosuccinimidyl 4-(N-maleimidomethyl)cyclohexane-1-carboxylate (Sulfo-SMCC; Pierce, Rockford, IL, USA). The array printing was performed by using a Telechem Nanoprint60 (Arrayit, Sunnyvale, CA, USA) hat spotted approximately 500 pL of 1 mg/mL peptide per feature on glass slides with 48 Telechem series SMP2-style 946 titanium pins (Arrayit).

### 2.3. Formaldehyde Crosslinking

Firstly, 20 μM PEP-1 (sequence of “RVFKRKRWLHVSRYYFGSC”) was incubated with 30 nM β-Gal for 20 min, and then 0.5% formaldehyde was added to the peptide/enzyme mixture. It was then incubated for another 20 min in order to crosslink the peptide with enzyme. The entire mixture was then diluted by 100 × in phosphate buffer to bring the overall peptide concentration as low as 200 nM.

### 2.4. Microarray Binding

Binding of enzymes on the microarray was performed as previously described [17,18]. Briefly, a microarray was first pre-washed with surface cleaning solvent (7.33% (*v*/*v*) acetonitrile, 37% isopropyl alcohol, and 0.55% trifluoroacetic acid in water), followed by the treatment of a capping buffer (3% (*v*/*v*) bovine serum albumin (BSA), 0.02% (*v*/*v*) mercaptohexanol, 0.05% (*v*/*v*) Tween 20 in 1 × PBS) to block any active SMCC linker on the array surface. Then, the array was incubated with a solution containing 5 nM Alexa 647-labeled β-Gal or PEP-1–β-Gal complex for two hours, allowing the enzyme to bind with peptides on the array surface. After washing off unbound enzymes, the array was read by a standard array reader (PerkinElmer, Waltham, MA, USA) with color scanning using 647 nm laser lines.

### 2.5. Enzyme Inhibition Assays

Solution-based enzyme assays were performed on SpectraMax M5 96 well plate readers (Molecular Device, Sunnyvale, CA, USA) as described previously [17]. Simply explained, peptides were first incubated with enzyme for 20 min, and then the substrate solution was added into the wells to measure the enzyme activity. At least three replicates per peptide were included. The β-gal-catalyzed hydrolysis of RBG was fluorescently monitored at 590 nm (resorufin) with the excitation at 540 nm. For a typical assay, ~0.3 nM or 1 nM β-Gal was incubated with peptides and 100 μM RBG substrate in pH 7.4, 10 mM potassium phosphate buffer with 100 μM MgCl_2_ at 25 °C. The reaction rate was determined by the initial velocity of the linear reaction. The percentage of reduced/inhibited enzyme activity was calculated as below:

$$\text{Percentage of reduced activity}=\left(1-\frac{\text{Inhibited activity}}{\text{Noninhibited activity}}\right)\times 100\%$$

For recovery of PEP-1 inhibited enzyme activity, 1 nM β-Gal was first incubated with PEP-1 for 20 min for inhibiting activity, and then NEG peptide was added to the solution and incubated for another 20 min before the activity assay.

## 3. Results and Discussion

**Figure 1:**
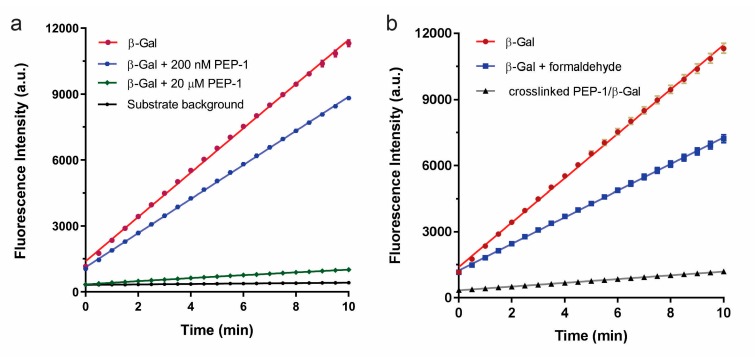
Enhancement of inhibition by crosslinking peptide with enzyme. (**a**) PEP-1 (“RVFKRKRWLHVSRYYFGSC”) decreased the inhibition of β-galactosidase (β-Gal) when it was diluted from 20 μM (green) to 200 nM (blue); (**b**) Formaldehyde-crosslinked PEP-1–β-Gal complex (green) with strongly inhibited enzyme activity, even at 200 nM peptide concentration.

We previously published an approach of selecting enzyme inhibitors from a hydrogel-coated microarray composing of 10,000 20-mer random peptides [17]. PEP-1 was found to inhibit β-Gal with an IC_50_ (half maximal inhibitory concentration) value ~1.6 μM (supplementary Figure S1). PEP-1 is a positively charged peptide with a isoelectric point (pI) ~11, and thus it can electrostatically bind to the negatively-charged surface of β-Gal (pI ~4.6) at physiological pH. Further kinetic study suggested that PEP-1 inhibited β-Gal in a mixed model with an increased *K_m_* and a reduced *k_cat_* (unpublished result). The aggregation between PEP-1 and β-Gal might primarily result in the inhibited enzyme activity. To stabilize PEP-1–β-Gal complex with inhibited enzyme activity, we used formaldehyde to covalently crosslink the peptide with the enzyme surface that permanently inhibited enzymes. As shown in Figure 1a, without formaldehyde crosslinking, PEP-1 decreased the ability to inhibit β-Gal when its concentration was diluted from 20 μM to 200 nM. Conversely, the activity of the crosslinked PEP-1–β-Gal complex was still strongly inhibited at 200 nM peptide concentration as compared with native enzymes (Figure 1b). This suggested that β-Gal was almost irreversibly inhibited by crosslinking the inhibitory peptides with enzyme. As a control, the mixture of β-Gal and formaldehyde was much more active than the crosslinked PEP-1/β-Gal complex, suggesting that formaldehyde crosslinking did not significantly damage the enzyme activity under the experimental condition (maintained >50% activity).

**Figure 2:**
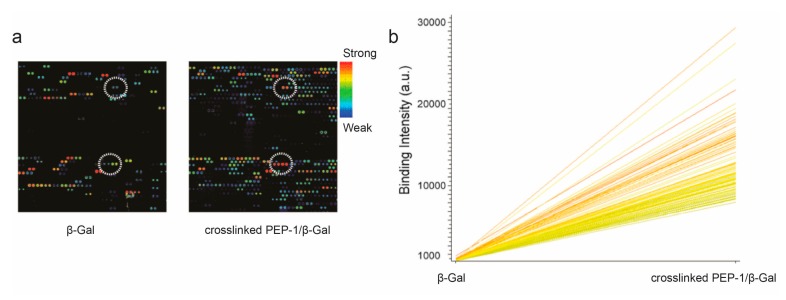
Selection of new peptides binding to the crosslinked PEP-1–β-Gal complex on microarray. (**a**) Fluorescent scanning images (a representative region) of peptide binding for β-Gal (left) and crosslinked PEP-1–β-Gal complex (right). β-Gal was labeled with Alexa Fluor 647 (Alexa 647). The representative peptide spots were circled that showed the increased binding of PEP-1-β-Gal complex. (**b**) The top peptides that bound more strongly to crosslinked PEP-1–β-Gal than to β-Gal.

**Figure 3:**
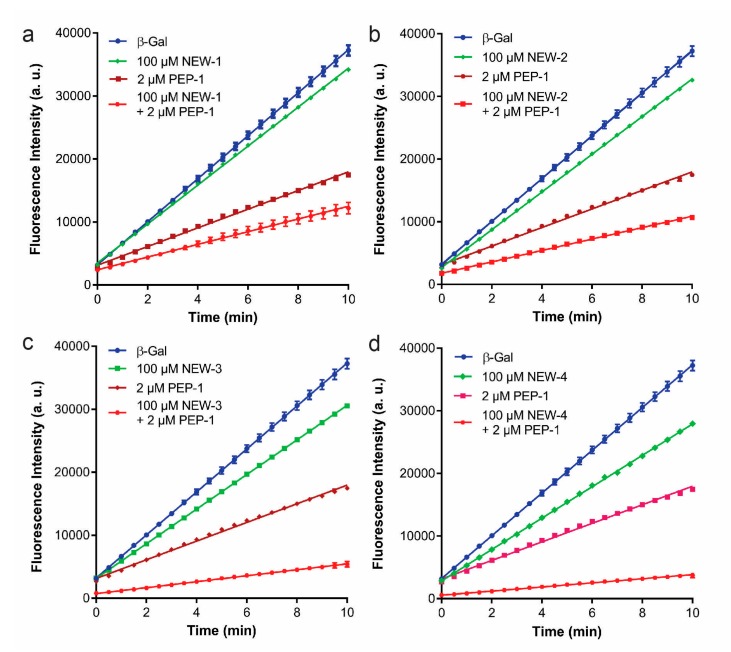
Test of new selected peptides that were paired with PEP-1 for inhibiting β-Gal. (**a**) NEW-1; (**b**) NEW-2; (**c**) NEW-3; and (**d**) NEW-4.

To search for new peptides that only bound to the inhibited PEP-1–β-Gal complex, we first incubated the solution of crosslinked PEP-1–β-Gal complex onto the microarray composed of 10,000 20-mer random peptides. When compared to the array profiles of peptides binding to β-Gal, multiple peptides showed increased binding intensity for crosslinked PEP-1–β-Gal (Figure 2a). Figure 2b showed the top 100 peptides that showed more binding intensities for crosslinked PEP-1/β-Gal than that for individual enzymes on microarray. We hypothesized that the peptides that only recognized the PEP-1–β-Gal complex without strong binding of β-Gal, could potentially be paired with PEP-1 to enhance the inhibition of β-Gal. To test this hypothesis, we selected four new peptides that bound weakly to β-Gal, but showed a more than 30-fold increase of binding intensity for PEP-1–β-Gal complex (Table 1). Four newly-selected peptides (NEW-1–4) were synthesized and tested for enhancement of the inhibition of β-Gal with the addition of PEP-1. As shown in Figure 3a, 100 μM NEW-1 inhibited β-Gal very weakly with less than 10% reduction of enzyme activity. However, the combination of 100 μM NEW-1 and 2 μM PEP-1 produced more inhibition of β-Gal (~70% reduction) than that for PEP-1 by itself (~57% reduction). As shown similarly in Figure 3b, the combination of 100 μM NEW-2 and 2 μM PEP-1 inhibited ~73% of β-Gal activity. This amounted to more inhibition than that for NEW-2 (~12% reduction) and PEP-1 (~57% reduction) at the same peptide concentrations. NEW-3 and PEP-1 were paired together to inhibit ~86% of enzyme activity. In contrast, NEW-3 by itself only inhibited ~19% of enzyme activity (Figure 3c) at the same peptide concentration. As shown in Figure 3d, the combination of NEW-4 and PEP-1 produced a 91% inhibition of β-Gal activity, which was a much stronger inhibition than that using either NEW-4 (~26% reduction) or PEP-1 (~57% reduction) alone. Based on above results, all four selected peptides showed the enhancement of β-Gal inhibition when they were used together with PEP-1. The combination of NEW-3 or NEW-4 with PEP-1 produced more enzyme inhibition than that produced by NEW-1 or NEW-2 paired with PEP-1. These results indicated that the cooperative pair of inhibitory peptides could be selected from microarray using our developed approach.

**Figure 4:**
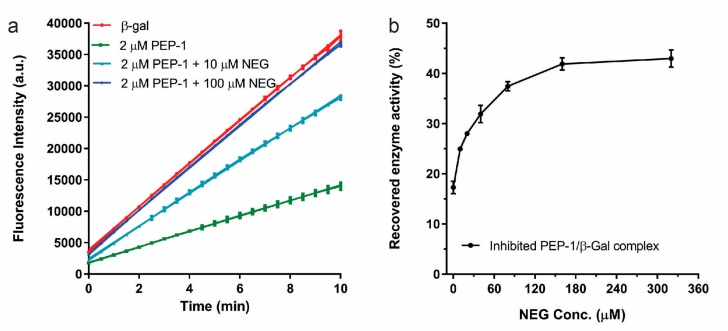
(**a**) The reduction of the enzyme inhibition by mixing PEP-1 with the negative control peptide of NEG. (**b**) The recovery of the inhibited PEP-1–β-Gal complex was achieved by adding NEG peptide.

As a negative control, we also selected a peptide that neither bound to β-Gal nor inhibited the PEP-1–β-Gal complex. NEG peptide (“ESVPTDLPMDTMEGKNWGSC”) was an overall negatively-charged peptide with a low pI value of ~3.7. As shown in Figure 4a, the combination of 10 μM NEG with 2 μM PEP-1 only inhibited 24% of β-Gal activity, as compared with 57% activity reduction with 2 μM PEP-1 by itself. The mixture of 100 μM NEG and 2 μM PEP-1 almost lost the ability to inhibit β-Gal. In Figure 4b, the addition of NEG peptide also recovered the inhibited activity of PEP1–β-Gal complex. The activity of inhibited PEP-1–β-Gal complex was increased from less than 20% of uninhibited enzyme to nearly 45% of uninhibited enzyme by adding 0 to 320 μM NEG peptide. It was likely that negatively-charged peptides could disrupt the interaction between positively-charged PEP-1 (pI~11) and β-Gal, and destabilize the inhibited PEP-1–β-gal complex. Thus, our control NEG peptide did not show any cooperatively enhanced inhibition with PEP-1, and could also recover the activity of inhibited peptide/enzyme activity. Other negatively-charged peptides (e.g., “EFSNPTAQVFPDFWMSDGSC”) were also observed to have a similar effect of recovering the activity of inhibited PEP-1–β-gal complex.

## 4. Conclusions

In summary, we developed an approach for selecting cooperative peptide pairs for inhibiting enzymes on screening an array of peptides. The primary peptide inhibitor was crosslinked with enzymes to stabilize the inhibited peptide–enzyme complex. Then, secondary effect peptides were identified as only binding strongly to the inhibited peptide–enzyme complex. These secondary effect peptides also bound weakly to uninhibited enzyme on microarray, and thus had little effect on enzyme activity in solution by themselves. We validated the selected secondary effect peptides in a solution-based enzyme assay in which the combination of primary peptides and secondary effect peptides produced more inhibition on enzyme activity than that for primary peptides or secondary effect peptides alone. For negative control, we also tested a negatively-charged peptide that did not show strong binding to β-gal or crosslinked PEP-1–β-gal complex. As a result, this NEG peptide not only showed no cooperative inhibition with PEP-1, but also disrupted the inhibited PEP-1–β-gal complex, recovering enzyme activity. The selection of these cooperative or noncooperative peptide pairs may provide a versatile toolkit for modulating enzyme functions, which may find more utility in drug discovery and biocatalysis. A similar approach for searching enzyme modifiers could also be applied to small molecules that are capable of array.

## Figures and Tables

**Table 1:** Selected peptides that showed increased binding to the PEP-1–β-Gal mixture, as screened using peptide microarrays.

Peptide	Sequence	β-Gal Binding ^1^ (a.u.)	PEP-1/β-Gal Binding ^2^ (a.u.)
NEW-1	GVSHLHWIKMLNETTVMGSC	486.8 ± 81.3	27,417.3 ± 4933.1
NEW-2	HISPQHMMAYSPKAFDYGSC	301.0 ± 53.8	21,378.3 ± 4007.7
NEW-3	YDTLHRNRQMMDWQFEPGSC	334.7 ± 42.9	25,598.5 ± 2407.7
NEW-4	MHNHAFNDNHGRGPTAWGSC	1210 ± 184.6	30,398.0 ± 3998.7

^1^ The microarrays were incubated with 5 nM Alexa 647-labeled β-Gal. ^2^ The peptide microarrays were incubated with a solution containing 5 nM crosslinked Alexa 647-labeled β-Gal/PEP-1.

## Supplementary Materials

The following are available online at www.mdpi.com/link/2076-3905/6/2/8/S1, Figure S1: Titration of IC_50_ for PEP-1.
