# Supplementary material for: Microarray Selection of Cooperative Peptides for Modulating Enzyme Activities
Source: Microarrays (Basel). 2017 Apr 26;6(2):8. doi: 10.3390/microarrays6020008 (PMC5487955; doi:10.3390/microarrays6020008)
Supplement: Supplementary file 1 [file microarrays-06-00008-s001.pdf]

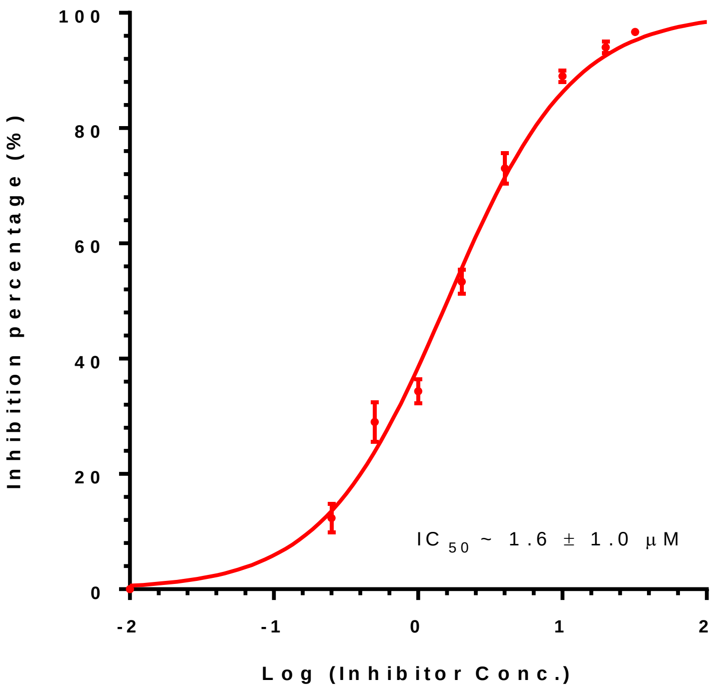

Figure S1. Titration of IC_{50} for PEP-1. The IC_{50} of peptide inhibition is measured at a substrate concentration of 100 μM RBG (resorufin β-D-galactopyranoside) and β-Gal concentration of 150 μg/L, 25 °C. All tests included three replicates. Error bar: range of data.

The IC_{50} of each inhibitor was determined by fitting the concentration vs. inhibition curve to the function 'Fit LogIC50' as defined in the program GraphPad Prism 7 using the fitting equation "Y=Bottom+(Top-Bottom)/(1+10^(X-LogIC50))". The "Bottom" term was constrained to 1, which represents the maximal inhibition of 100%. The "Top" term was constrained to 0, which represents the minimal inhibition of 0%.

The enzyme inhibition was also quantitatively characterized using the inhibition percentage that was calculated using the following equation:

Inhibition Percentage = $(\text{Activity}_{\text{uninhibited}} - \text{Activity}_{\text{inhibited}}) / \text{Activity}_{\text{uninhibited}} \times 100\%$
